# The Lumbosacral Erector Spinae Plane Block for Abdominal Hysterectomy: A Case Report

**DOI:** 10.7759/cureus.72705

**Published:** 2024-10-30

**Authors:** Francesco Marrone, Tommaso Sorrentino, Pierfrancesco Fusco, Maria Eleonora Monte, Saverio Paventi, Marco Tomei, Fabrizio Fattorini, Carmine Pullano

**Affiliations:** 1 Anesthesia, Santo Spirito Hospital, Rome, ITA; 2 Faculty of Medicine, Cattolic University Rome, Rome, ITA; 3 Faculty of Medicine, Sapienza University Rome, Rome, ITA; 4 Anesthesia, San Giovanni di Dio Hospital, Crotone, ITA; 5 Anesthesia and Intensive Care Unit, San Filippo e Nicola Hospital, Avezzano, ITA; 6 Anesthesia, Magna Graecia University, Catanzaro, ITA; 7 Faculty of Medicine, Sapienza University of Rome, Rome, ITA; 8 Anesthesiology and Critical Care, Santo Spirito Hospital, Rome, ITA; 9 Anesthesiology, Sapienza University Rome, Rome, ITA; 10 Anesthesia, Villa Pia Clinic, Rome, ITA

**Keywords:** analgesia, fascial blocks, hysterectomy, postoperative pain, sacral erector spinae

## Abstract

After major abdominal surgery and open hysterectomy, postoperative pain management is often challenging. Various abdominal fascial and truncal blocks, including paravertebral, erector spinae plane, transversus abdominis plane, and quadratus lumborum blocks, have been evaluated for their efficacy. When used in a multimodal pain control strategy, after an open abdominal hysterectomy under spinal anesthesia, the novel sacral erector spinae plane block showed promising results in terms of safety, efficacy, and minimal invasiveness.

## Introduction

After cesarean section (C-section), hysterectomy represents the most common surgical procedure in women for the management of an enlarged or bulky uterus. Postoperative abdominal pain following abdominal hysterectomy can often be of significant intensity, posing a detrimental factor to postoperative recovery and prolonging hospital stays. Adequate perioperative analgesia is crucial not only for enhancing patient comfort but also for facilitating early mobilization, reducing the risk of thromboembolism, and shortening hospital stay after the procedure [1].

Various abdominal truncal blocks, such as paravertebral block, erector spinae plane (ESP) block, transversus abdominis plane (TAP) block, and quadratus lumborum (QL) block, have been described in the literature, each with varying degrees of efficacy in managing postoperative pain in adult abdominal surgery [2]. In a total abdominal hysterectomy, few studies have examined single-injection regional analgesia techniques other than the TAP block and wound infiltration. Among them, ESP block and QL block seemed to prove superior analgesic effects [3].

Here, we describe the case of an obese patient who underwent an open hysterectomy, for which we integrated a single-injection, modified lumbosacral (LS)-ESP block to provide postoperative pain management. The use of this approach aimed to produce effective analgesia, minimizing the reliance on systemic opioids and facilitating the patient’s early mobilization and recovery. This case highlights the potential benefits of incorporating regional anesthetic techniques, such as the (sacral) ESP block in a multimodal pain treatment regimen. The CARE guidelines for reporting clinical cases were followed in presenting this case report [4].

## Case presentation

An obese (weight, 90 kg; height, 160 cm; body mass index, 35 kg/m^2^) American Society of Anesthesiologists-Physical Status III patient with thoracolumbar scoliosis and a left-sided thoracic hump, allergy to non-steroidal anti-inflammatory drugs (NSAIDs) (anaphylactic shock with diclofenac, rash and angioedema with ketorolac, and urticaria and rash with etoricoxib), intolerant to opioids (nausea, vomiting, itching, and dizziness) during previous orthopedic procedures underwent an open abdominal hysterectomy for bulky uterus under spinal anesthesia. A modified LS-ESP block was administered for analgesic purposes as part of a multimodal treatment regimen. Epidural catheter analgesia was not considered due to the patient’s refusal.

**Figure 1:**
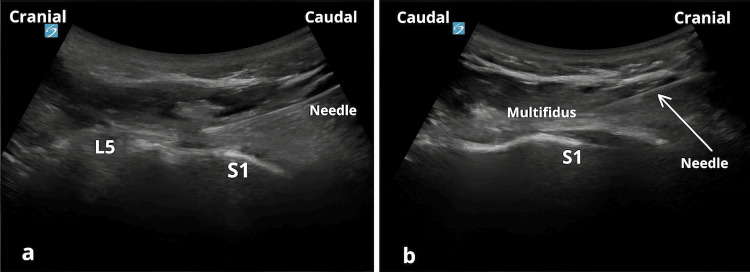
The modified lumbosacral erector spinae plane block. a: The caudocranial approach of the needle from S1 toward the L5 lamina. b: Reverse approach of the needle, craniocaudal toward S1.

After obtaining patient consent, the sacral ESP block was performed aseptically and under vital parameter monitoring with an injection at the sacral median crest, S1 level, using a dynamic approach and a double craniocaudal/caudocranial injection, at the same puncture point, verifying the spread of the injected solution toward the sacrococcygeal joint as well as toward the L5 and L4 laminae (Figure 1).

A solution comprising 0.18% ropivacaine 30 mL, dexamethasone 4 mg, and dexmedetomidine 15 µg was administered. The block was tested with a cold wet cotton swab and needle prick after 15 and 25 minutes, checking its maximum extension from the iliac crests to the thighs, including the perineum, pubis, and gluteal area (L2-S4). Then, the patient was brought to the operating room where spinal anesthesia in aseptic conditions was placed at the T12-L1 level using a 25-G spinal needle along with ropivacaine 8 mg. Surgery lasted 71 minutes and was uneventful. Vital parameters were monitored. We planned a scheduled analgesic regimen and the potential use of tramadol in the postoperative period if the Numerical Rating Scale score was >5. However, the patient did not need on-clock analgesic administration, and opioids were never administered. Only 1 g of intravenous paracetamol was administered after 12 hours of the operation. The patient was discharged from the hospital on the third day after surgery, without complications.

## Discussion

Three randomized controlled trials have already proven the effectiveness of bilateral low thoracic (T9) ESP block on postoperative pain after abdominal hysterectomy. They showed reduced (albeit not abolished) postoperative opioid usage with acceptable postoperative analgesia up to 24 hours after the procedure [5-7].

The novelty of this case report is the use of a modified approach to ESP block, starting from a sacral median crest (S1), in a technically simpler mode (single puncture), with a more easily reachable target (sacral bone), even for obese individuals, and with reduced potential complications (the target area in the sacral ESP block is far from large vessels and nerves). We hypothesized that for procedures involving the lower abdomen, such as open hysterectomy, pelvic pain would be predominant. At the same time, the somatic component related to the abdominal wall and the surgical wound (Pfannenstiel incision in our case) would have less impact.

We placed a modified LS-ESP block targeting the sacral interfascial plane under the multifidus muscle aponeurosis and the lumbar erector spinae muscle by using a single injection and dynamic technique to achieve the hydrodissection effect in the opening of the fascial planes [8].

The mechanism of action of the sacral ESP block is still considered speculative. Anatomical studies [9,10] and numerous clinical experiences in many surgical contexts, including orthopedics [11] suggest that this block is not merely an analgesic block of the posterior branches of the sacral spinal nerves. It may also have effects (even not entirely predictable) on the sacral and lumbar plexus roots due to the cranial and anterior diffusion of the injected solution starting from the fascial plane of the sacral multifidus aponeurosis on the dorsal surface of the sacrum.

The modified LS-ESP block may better work in postoperative pain management after abdominal hysterectomy (while potentially not in postoperative C-section pain) because the uterus is removed, and what is no longer there, does not hurt anymore. After the removal of the organ, pain is primarily pelvic (vaginal fornices) and the surgical wound remains exposed. Patients who require more surgical dissection and manipulation might experience greater postoperative visceral discomfort. We argue that the modified LS-ESP block may effectively manage postoperative pain, as this block effectively manages pelvic pain. Nevertheless, although our report does not prove that a low thoracic/lumbar ESP block would not have been equally effective, it is noteworthy that the patient did not use opioids or other analgesics on a fixed schedule and requested only a single dose of paracetamol in 48 hours.

## Conclusions

In our experience, the modified LS-ESP block, with better targeting of pelvic pain after an open hysterectomy, was effective, safe, and minimally obtrusive. Further studies are warranted to gain a deeper understanding and define better strategies in this specific context.
